# Slovak Translation and Cross-Cultural Validation of the Eating Assessment Tool (EAT10^®^)

**DOI:** 10.3390/jcm11195966

**Published:** 2022-10-10

**Authors:** Zofia Frajkova, Dimitrios Paouris, Ladislav Nado, Ivana Vyrvova, Adelaida Fabianova, Athanasia Printza, Lukas Varga, Miroslav Tedla

**Affiliations:** 1 Department of Otorhinolaryngology and Head and Neck Surgery, Medical Faculty, Comenius University, 85101 Bratislava, Slovakia; 2 Department of Communication Disorders, Faculty of Education, Comenius University, 83102 Bratislava, Slovakia; 3 Department of Neurology, Faculty Hospital, 91775 Trnava, Slovakia; 4 Department of Pediatric Otorinolaryngology, Medical Faculty and National Institute of Children’s Diseases, Comenius University, 83101 Bratislava, Slovakia; 5 VH Analytics, 96205 Hriňová, Slovakia; 6 Axis Medical Center, 92101 Piešťany, Slovakia; 7 1st Otolaryngology Department, Medical School, Faculty of Health Sciences, Aristotle University of Thessaloniki, Thessaloniki 54124, Greece; 8 DIABGENE Laboratory, Biomedical Research Center, Slovak Academy of Sciences, 83101 Bratislava, Slovakia; 9 Institute of Cancer and Genomic Sciences, University of Birmingham, Birmingham B15 2SQ, UK

**Keywords:** eating, swallowing disorders, oropharyngeal dysphagia, subjective assessment, EAT10^®^, psychometric properties, Slovakia

## Abstract

Introduction: The objective of the study was the language adaptation and verification of psychometric properties of the Slovak version of the EAT10^®^ questionnaire. Methods: The original English version of the questionnaire was translated into the Slovak language. The research group included 136 control participants and 51 dysphagic patients. Test–retest reliability, item analysis, internal consistency, construct and clinical validity, and Receiver Operating Characteristic (ROC) analysis were performed to verify the psychometric properties of the Slovak EAT10^®^. Results: The internal consistency assessed with Cronbach’s alpha is excellent (α = 0.94). Statistical analysis of the Slovak version of EAT10^®^ showed excellent reliability (0.91, *p* < 0.001) in the test–retest. Through item-to-total correlation, we found out that all items significantly correlated with the overall score in EAT10^®^. Factor analysis proved a high construct validity. The EAT10^®^ questionnaire was able to reveal a latent variable: a swallowing disorder, which was affecting a group of patients. The clinical validity results confirmed statistically significant differences in the mean scores of the control and dysphagic groups (z = −10.30; *p* < 0.001). By dividing the dysphagic group into four subgroups (Head and Neck Cancer, Extraesophageal Reflux, Iatrogenic, and Neurological) there were nonsignificant differences in the mean scores of the subgroups. The cut-off value for the Slovak EAT10^®^ is three points. Conclusion: The Slovak EAT10^®^ is a valid and reliable tool designed for the subjective assessment of oropharyngeal dysphagia in patients.

## 1. Introduction

A swallowing disorder is a pathological condition commonly caused by various diseases, including neurological, otorhinolaryngological, and geriatric diagnoses [1,2]. However, the rate of prevalence of swallowing disorders in Slovakia cannot be accurately determined, due to insufficient and inconsistent reports of dysphagia based on screening, clinical and instrumental examination, and the lack of valid tools for determining its occurrence in Slovakia.

Assessment of swallowing disorders is a complex of subjective and objective evaluations made to consider the overall clinical conditions of a patient. In addition to the evaluation performed by the healthcare professional, the patient’s self-assessment should be a part of complex healthcare in order to increase interest in patient needs [3]. It is most often implemented using questionnaires in which the patient expresses his/her opinions, attitudes, and the impact of the disease on their life. The impression of the state of health is partially individual, based on the patient’s feelings and thoughts [4].

Two types of evaluation from the patient’s point of view are distinguished: the evaluation of health-related quality of life (HRQoL) and functional health status (FHS) [5]. The Eating Assessment Tool (EAT10^®^) is used to assess functional health status, focusing on swallowing. A patient quantifies the severity of their symptoms of swallowing disorders on a self-assessment scale [6].

The subjective swallowing assessment by the patient helps to identify the key mechanisms that the patient recognizes as important in relation to swallowing disorders [4,7]. Patients with the same diagnosis may evaluate the impact of the swallowing disorder in a different way [8]. The self-perception of swallowing is not always related to the severity of dysphagia and each patient perceives their swallowing disorder differently [9,10,11,12]. Self-assessment tools should be a part of comprehensive healthcare to improve treatment outcomes [3]. Currently, there are a number of questionnaires and other tools for assessing health from a patient’s perspective [6]. It is important to consider which evaluation tool to choose, depending on the objective of the evaluation.

EAT10^®^ was developed in 2008 and is one of the most frequently used self-assessment questionnaires in patients with swallowing disorders [13]. The adaptation of questionnaires in different countries of the world requires verification of psychometric properties. Incorrect or insufficient adaptations can lead to false data and incorrect conclusions [14]. In Slovakia, a valid tool for the assessment of swallowing from the patient’s point of view has not yet been adapted. We chose the EAT10^®^ tool for its simplicity, comprehensibility, and broad use in both clinical and research settings. The objective of the study was the linguistic adaptation of EAT10^®^ to the Slovak language and verification of its psychometric properties. The partial goals of the study were to assess the internal consistency, the reliability measured via test–retest reliability, the item-to-total correlation, and the construct validity. Validity analysis evaluated the differences between the scores of dysphagic patients divided into subgroups. ROC analysis was performed to determine the cut-off score, sensitivity, and specificity in capturing individuals with possible manifestations of oropharyngeal dysphagia. The COSMIN checklist was used in all evaluations relevant to the present study, including cross-cultural validity, internal consistency, reliability, and construct validity [15].

## 2. Materials and Methods

### 2.1. Linguistic Adaptation and Data Collection

The original version of the questionnaire [13] was translated from English to the Slovak language by two bilingual experienced professionals (an ENT specialist and a speech and language pathologist). Each of the experts translated the items independently, maintaining conceptual and linguistic equivalence with the original version [13]. Based on a consensus between the two experts, the controversial items were unified. There was no need to change or eliminate part of the instrument as part of cultural adaptation. This draft was translated into English by an independent bilingual English/Slovak speaking person who had no knowledge about the questionnaire. The Slovak version of the questionnaire together with the back-translated English version was sent to the first author of the original questionnaire, who approved the intention to adapt the Slovak version of the EAT10^®^ questionnaire by e-mail communication and signing the license agreement. He had no additional comments on the translated versions. The final translated version of the Slovak EAT10^®^ was created (Table 1).

### 2.2. Procedure

The intention to verify the Slovak version of EAT10^®^ was approved by the Ethics Committee of the University Hospital Bratislava in May 2019. Individuals were invited to participate between September 2019 and April 2020 by the Department of Otorhinolaryngology and Head and Neck Surgery, Comenius University and University Hospital Bratislava. All participants were recruited voluntarily. The purpose of the study was explained to the participants in advance, and all provided written informed consent. Each participant in the study filled in the EAT10^®^ questionnaire without the help of the administrator.

### 2.3. Participants

The control group inclusion criteria were: (1) aged over 18 years, (2) no history of head and neck surgery, and (3) no history of neurological diseases. Participants were recruited to the study from the general population. The dysphagic group inclusion criteria were: (1) aged over 18 years and (2) oropharyngeal dysphagia confirmed by a clinician (ENT specialist, neurologist, and speech and language pathologist) based on a clinical bedside swallowing examination and instrumental assessment (FEES—Flexible Endoscopic Evaluation of Swallowing).

Demographic characteristics of the participants including sex, age, and BMI variables for both the control and dysphagic group can be seen in Table 2. The control group consisted of 136 respondents: 67 women and 69 men between 20 and 81 years of age (mean 56.68 years of age). Fifty-one adult Slovak patients diagnosed with oropharyngeal dysphagia were enrolled: 34 women and 17 men between 20 and 91 years of age (mean 58.02 years of age).

### 2.4. Statistical Analysis

Statistical analysis was performed to confirm that the control and dysphagic groups were similar at baseline, i.e., with respect to age, BMI, and gender ratio. Due to significant deviations from the Gaussian distribution in age and BMI values, we applied the Wilcoxon rank-sum test. To compare gender ratios, Pearson’s χ^2^ test was used. Several statistical methods were used to measure internal consistency, reliability, construct validity, composite reliability, and clinical validity of EAT10^®^.

Internal consistency was measured using Cronbach’s alpha on the full dataset (*n* = 187), on datasets divided into groups (control and dysphagic), and also on 10 subsets made by removing a single item of the 10 items (i.e., α if item removed). Obtained values were interpreted according to recommendations as follows: α between 0.70 and 0.80 indicated satisfactory, between 0.8 and 0.9 indicated good, and greater than 0.9 indicated excellent consistency [16,17].

Test–retest reliability was assessed using Spearman’s correlation coefficient separately for each item and also for a total score. The results of the first and second questionnaire administration were compared [18]. No clinical intervention occurred during the 3 weeks between the test and retest. A correlation value of 1 indicates an excellent correlation, and a value close to 0 indicates low tool viability [19]. Additionally, to evaluate the performance of items, item-to-total correlation was calculated. To interpret correlation coefficients, the protocol by Altman (1991) was used. It considers r_s_ < 0.4 as weak, r_s_ = (0.4–0.6) as average, r_s_ = (0.61–0.8) as good, and r_s_ > (0.81–1.0) as excellent reliability [20].

Construct validity and composite reliability were analyzed in this study. A confirmatory factor analysis (CFA) was used to infer whether the Slovak EAT10^®^ is able to reliably measure the intended construct (the latent variable is oropharyngeal dysphagia). The following indicators were used to diagnose the CFA model: (i) RMSEA (root mean square error of approximation), in which the limit for an acceptable model is a value lower than 0.06 [21], (ii) SRMR (standardized root mean square residual), in which the limit for an acceptable model is a value lower than 0.08 [22], and (iii) CFI (comparative fit index), in which the limit for an acceptable model is a value higher than 0.95 [22]. Subsequently, standardized weights of individual factors were extracted from the CFA model and used to calculate the index of composite reliability (CR), in which values greater than 0.9 indicate high reliability. The construct validity analysis described above was performed for the full dataset (*n* = 187) and also partially for two datasets: the control group (*n* = 136) and the dysphagic group (*n* = 51).

In addition to these reliability measures, the Wilcoxon rank-sum test was used to verify the clinical validity of the Slovak EAT10^®^. The test explores the difference in obtained total scores between the control and dysphagic groups. It was further investigated whether it is possible to determine different types of dysphagia according to the total Slovak EAT10^®^ score. In this analysis, the four groups of diagnoses were compared according to the cause—HNC, esophageal, iatrogenic, and neurogenic—using Kruskal–Wallis one-way analysis of variance.

Finally, a ROC curve was calculated to describe the quality of the binary classifier (presence/absence of dysphagia) depending on the setting of its classification threshold (total EAT10^®^ score). The sensitivity and specificity values capturing test accuracy were calculated from the ROC analysis. In addition, an AUC index providing an aggregate measure of performance across all possible classification thresholds was determined. The cut-off score was calculated. It is the identification of the optimal threshold value for the overall EAT10^®^ score at which the diagnosis of dysphagia has the highest possible sensitivity and specificity at the same time. The bootstrapping method (n_bootstrap_ = 1000) was used to evaluate a more thorough estimate of possible classification thresholds, which were then visualized with a density plot.

Data handling and all calculations were performed in the R 3.6.3 environment for statistical computing [23] using the packages “psych” [24], “psychTools” [25], “coin” [26], and “lavaan” [27].

## 3. Results

The control and dysphagic groups were statistically equal with respect to age (z = −0.673, *p* = 0.501) and the gender ratio (χ^2^ = 3.80, df = 1, *p* = 0.051). The only difference found was that individuals in the dysphagic group had significantly lower BMI values (median = 23.27) in comparison to the control group (median = 26.03, z = 2.872, *p* = 0.004). Descriptive values are given in Table 2.

### 3.1. Internal Consistency

The value of Cronbach’s alpha was 0.94 (with a 95% confidence interval from 0.93 to 0.95). Calculated separately for the control group the α = 0.89 (95% CI: 0.86–0.91) and for the dysphagic group the α = 0.87 (95% CI: 0.81–0.92), which strongly indicates almost excellent consistency of the Slovak EAT10^®^. Values calculated from the dataset reduced by any single item (i.e., “α if item deleted” in Table 3) were not lower than 0.93.

### 3.2. Test–Retest Reliability and Item-to-Total Correlation

Test–retest reliability and item-to-total correlation indicate high levels of repeatability and reliability, perhaps with the exception of item no. 6: “Swallowing is painful.” Test–retest correlation in this item is almost equal to zero (r_s_ = −0.02; *p* = 0.896) and it has also a relatively low correlation to the total score of the Slovak EAT10^®^ (r_s_ = 0.56; *p* < 0.001).

### 3.3. Construct Validity

Confirmatory factor analysis revealed that the Slovak EAT10^®^ is able to measure latent variables, although RMSEA and CFI indicators do not support this notion in both stratified groups (RMSEA = 0.098; CFI = 0.08). SRMR indicates that this tool is highly valued when focused on the dysphagic group (SRMR = 0.08). Interpretation of construct reliability is, however, unclear: the control group indicates high reliability, but the Slovak EAT10^®^ performs poorly in the dysphagic group. If we focus on the parameters obtained from the whole dataset (*n* = 187) we conclude that according to SRMR (0.004) and CR (0.941) the performance of the Slovak EAT10^®^ is satisfactory.

### 3.4. Validity Analysis

**Figure 1:**
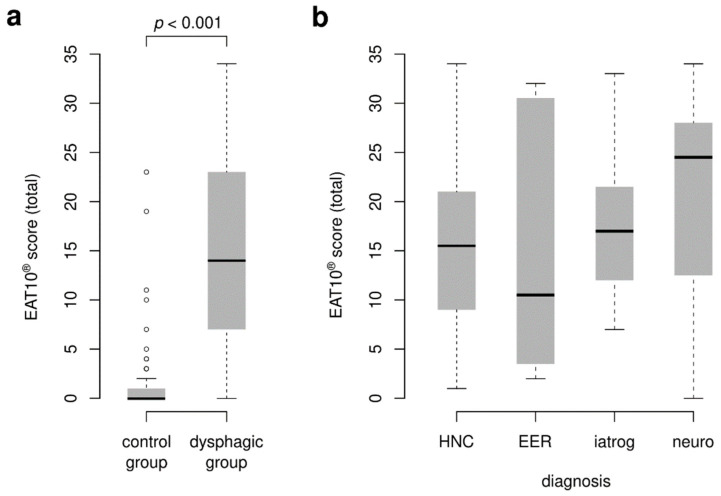
Clinical validity of EAT10^®^. Boxplots visualize the distribution of the total score values for (**a**) control and dysphagic groups of respondents and (**b**) dysphagic group of respondents based on the type of diagnosis. HNC = head and neck cancer; EER = extraesophageal reflux; Iatrog = iatrogenic dysphagia; Neuro = neurogenic dysphagia.

The Wilcoxon rank-sum test revealed significant difference between values of total scores obtained by the control (median = 0; range = 0–23) and dysphagic group (median = 14; range = 0–34; z = −10.30, *p* < 0.001; Figure 1a). However, no statistically significant difference in the total score was found between the various subgroups of dysphagia (Kruskal–Wallis χ^2^ = 1.69, d_f_ = 3, *p*-value = 0.638; Figure 1b). Thus, it is not possible to determine the cause of oropharyngeal dysphagia based on the overall EAT10^®^ score. Boxplots visualize the distribution of total score values for (a) control and dysphagic groups of respondents, and dysphagic subjects based on the type of diagnosis (b).

### 3.5. ROC Analysis

**Figure 2:**
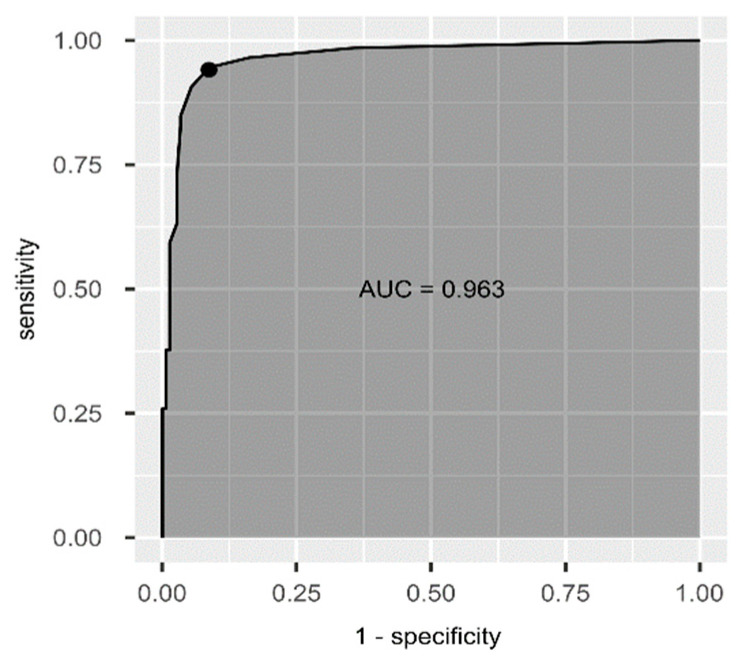
ROC curve of the Slovak EAT10^®^.

Based on the ROC analysis (Figure 2) we determined the AUC at 0.963 and also the cut-off score for the Slovak EAT10^®^. The optimal value estimated via the bootstrapping procedure is at three points (with a sensitivity of 94.12% and specificity of 91.28%).

## 4. Discussion

The EAT10^®^ questionnaire was designed in 2008 [13] and its original English version has been translated and adapted into more than 15 languages [16,17,18,19,20,21,22,23]. It is necessary to distinguish between the linguistic adaptation of the questionnaire and the verification of the psychometric properties of the questionnaire. In the case of the linguistic adaptation, the process of translation and back-translation is carried out in a precise manner [24,25]. The final consensus of all experts involved in the linguistic adaptation is needed to establish the final version of the questionnaire in a given language. The verification of psychometric properties is key to using an assessment tool in a given language. It is not sufficient to verify only the validity and reliability of the questionnaire [26]. The inconsistency in the verification of psychometric properties of translated questionnaire methods makes it difficult to reliably compare foreign versions.

This study demonstrated a very high internal consistency of the Slovak EAT10^®^. Similar results have been reported by foreign adaptations of EAT10^®^; Cronbach’s α ranged from 0.87 [27] to 0.963 [22,28,29]. The same Cronbach’s α as for the Slovak version of EAT10^®^ was calculated for the German adaptation of EAT10^®^ [30]. Based on the values of Cronbach’s α if an item is deleted, we conclude that the removal of any item will not cause an increase in the overall α value. The inclusion of all 10 items is, therefore, necessary and fully justified.

Reliability was assessed via test–retest reliability. Participants from the control group completed EAT10^®^ twice in three weeks. The selection of participants for the test–retest evaluation differs in the language versions. In the French [27] and Greek [22] adaptation of the EAT10^®^, a test–retest was performed by both the dysphagic and control groups. The Swedish adaptation reported that 13 participants in the control group performed a test–retest [19]. In the Arabic version of EAT10^®^, it was readministered to 14 patients within one to two weeks [17]. In the Slovak version of the EAT10^®^, the test–retest showed excellent reliability. Nevertheless, it should be emphasized that this high value was obtained from half of the EAT10^®^ items of the questionnaire. Adaptations in other languages show varying degrees of test–retest reliability ranging from r = 0.73 to r = 0.98 [17,22,31]. We hypothesize that such variability may be due to inconsistencies in the implementation of test–retest, especially the different times between the test and the retest, different groups of participants (control group vs. dysphagic group), and the cultural differences themselves.

The item-to-total correlation of the Slovak EAT10^®^ is statistically significant. This means that each item of the questionnaire increases the overall reliability of the tool [32]. The same results were demonstrated in the Hebrew and Arabic versions of EAT10^®^ [17,31]. The French, Greek, and Italian adaptations do not include item analysis [22,23,27].

Construct validity was evaluated using factor analysis. The validity of the latent variable was rejected for the control group. This rejection is logical, as the participants in the control group did not have the same diagnosis (a swallowing disorder in our case) that would connect them. Based on the constructed models of confirmatory factor analysis, we can conclude that the Slovak version of the EAT10^®^ questionnaire shows signs of high validity of the construct and is able to reveal a latent variable (a swallowing disorder). Defining construct validity is not a common part of linguistic adaptations of the EAT10^®^ questionnaire, therefore a comparison with other data is not feasible. In a study evaluating the validity of the EAT10^®^ construct using the RMSEA model, the results confirmed the unidimensionality of EAT10^®^ with two exceptions [33]. One of the four-factor analysis criteria to support EAT10^®^ unidimensionality was not met: the RMSEA value was higher than 0.08, which may be related to an insufficient research sample [33]. The second exception concerned two specific EAT10^®^ items that showed negative residual correlations indicating local dependence. This would mean another dimension of the questionnaire [33]. The results of construct validity in our study and other studies [26,33] support the unidimensionality of the EAT10^®^ questionnaire.

Based on calculations using the Wilcoxon test, a significantly higher score was confirmed in the dysphagic group in the Slovak version of the EAT10^®^ questionnaire compared to the control group. These results correlate with the results of the clinical validity of the original version of EAT10^®^ [13] and other linguistic adaptations of EAT10^®^ [17,22,27]. There is no reported study in which clinical validity has not been confirmed.

Our dysphagic group was divided into four subgroups according to the cause of dysphagia (HNC, EER, iatrogenic, neurological). There was no statistically significant difference in EAT10^®^ scoring between these groups.

When verifying the Italian version of EAT10^®^, three groups were compared: dysphagic, dysphonic, and control (asymptomatic) [23]. Statistically significant differences were calculated when comparing the scores of the dysphagic and dysphonic groups, and the dysphagic and control groups. When comparing the dysphonic and control groups, significant differences were found only in items 5, 8, and 9 [23]. The Greek adaptation of EAT10^®^ also includes a division into three groups [22], but the division is different from the Italian version [23]. In addition to the control and dysphagic groups, a group of participants with a dysphagia-related diagnosis (DRD) was included in the research. In this group were patients diagnosed with multiple sclerosis, chronic renal failure, neuromuscular diseases, patients after total laryngectomy, and other DRD. A significant main effect for the group (*p* < 0.001) was demonstrated for the total score and for single questions [22].

The EAT10^®^ questionnaire was not designed to screen swallowing disorders, it was created as a tool for self-assessment of swallowing function. However, several studies have indicated its use in the screening of swallowing disorders (Table 4).

By determining the cut-off value of the diagnostic tool, it is possible to distinguish a person with or without a certain disease/symptom. The accuracy of the test is stated by establishing sensitivity and specificity [35]. The cut-off value was calculated using ROC analysis. The cut-off for the Slovak version of EAT10^®^ is three points with a sensitivity of 94.12% and a specificity of 91.28%. Data from other studies indicate that the cut-off ranges from two to three points with varying degrees of sensitivity and specificity. The characteristics of the Slovak cut-off values, sensitivity, and specificity are most similar to the Swedish version [19].

### Strengths and Limitations of the Study

The strength of the study is the use of the COSMIN checklist as a standard protocol for linguistic adaptation and verification of the questionnaire’s psychometric properties. The limitation of the study is the smaller number of patients in the dysphagic group. Ongoing research should seek to determine patient groups in which the EAT10^®^ can accurately detect dysphagia and aspiration [36].

## 5. Conclusions

It was demonstrated that the Slovak version of the EAT10^®^ is characterized by good item-to-total correlation, internal consistency, reliability, validity, and repeatability. The Slovak EAT10^®^ is the first questionnaire evaluating the functional health status of patients with swallowing disorders verified in the Slovak language. The questionnaire can be used in the clinical process of diagnosing a swallowing disorder, or for readministration in a group of patients after swallowing therapy in Slovak-speaking patients.

## Figures and Tables

**Table 1:** The Slovak version of the Eating Assessment Tool (Slovak EAT10^®^).

Item	Original Version	Slovak Version
1	My swallowing problem has caused me to lose weight.	Kvôli mojim problémom s prehĺtaním som schudol/la.
2	My swallowing problem interferes with my ability to go out for meals.	Pre problémy s prehĺtaním nemôžem chodiť jesť do spoločnosti.
3	Swallowing liquids takes extra effort.	Prehĺtanie tekutín je pre mňa namáhavé.
4	Swallowing solids takes extra effort.	Prehĺtanie tuhej stravy je pre mňa namáhavé.
5	Swallowing pills takes extra effort.	Prehĺtanie tabliet je pre mňa namáhavé.
6	Swallowing is painful.	Prehĺtanie je bolestivé.
7	The pleasure of eating is affected by my swallowing.	Prehĺtanie ovplyvňuje môj pôžitok z jedla.
8	When I swallow food sticks in my throat.	Pri prehĺtaní sa mi jedlo zasekne v hrdle.
9	I cough when I eat.	Pri jedle kašlem.
10	Swallowing is stressful.	Prehĺtanie je pre mňa stresujúce.

**Table 2:** The demographic and clinical characteristics of the participants.

Variable	Value	Control Group		Dysphagic Group		Total	
		*n*	%	*n*	%	*n*	%
Gender	Female	67	49.3	17	33.3	84	44.9
	Male	69	50.7	34	66.7	103	55.1
Age	Mean ± SD	56.68 ± 13.74		58.02 ± 16.78		57.05 ± 14.6	
	Median	56		60		58	
	Min	20		20		20	
	Max	81		91		91	
BMI	Mean ± SD	26.75 ± 4.89		24.69 ± 5.28		26.19 ± 5.07	
	Median	26.3		23.27		25.5	
	Min	17.47		15.42		15.42	
	Max	45.91		43.15		45.91	
Diagnosis	HNC	-	-	12	23.5		
	Esophageal	-	-	8	15.7		
	Iatrogenic	-	-	7	13.7		
	Neurogenic	-	-	12	23.5		
	Other			12	23.5		

BMI = body mass index; *n* = number of participants; % = percentage; Mean ± SD = the average value ± standard deviation; HNC = head and neck cancer.

**Table 3:** Relative frequency (%) of answers to EAT10^®^ items and reliability parameters of the Slovak version of EAT10^®^.

Item	0	1	2	3	4	Test–Retest	Item-to-Total	α If Item Deleted
1	41.2	13.7	13.7	3.9	27.5	×	0.66 ***	0.93
2	41.2	5.9	11.8	15.7	25.5	×	0.69 ***	0.93
3	41.2	15.7	11.8	15.7	15.7	×	0.68 ***	0.93
4	27.5	19.6	11.8	13.7	27.5	0.81 ***	0.76 ***	0.93
5	33.3	19.6	15.7	13.7	17.6	0.91 ***	0.73 ***	0.94
6	54.9	21.6	15.7	3.9	3.9	−0.02	0.56 ***	0.94
7	29.4	17.6	31.4	11.8	9.8	×	0.74 ***	0.93
8	25.5	19.6	29.4	9.8	15.7	0.62 ***	0.75 ***	0.93
9	43.1	11.8	17.6	15.7	11.8	1 ***	0.68 ***	0.94
10	31.4	15.7	19.6	13.7	19.6	×	0.7 ***	0.93
Overall						0.91 ***		
Sample size						*n* = 58	*n* = 187	*n* = 187

Relative frequency (%) of answers to EAT10^®^ items and reliability parameters of the Slovak version of EAT10^®^. Values in the seventh and eighth columns are Spearman’s correlation coefficients, whereas the last column represents values of Cronbach’s alpha if the given item is removed from the dataset. Symbol × signifies items with null variability (all answers given by respondents were 0 = “no problem”). Asterisks denote the statistical significance.

**Table 4:** Cut-off values, sensitivity, and specificity values for foreign versions of EAT10^®^.

Authors	Language Version	Cut-Off	Sensitivity	Specificity
Belafsky et al., 2008 [13]	English	3	−	−
Schindler et al., 2013 [23]	Italian	3	−	−
Gonçalves et al., 2013 [18]	Brazilian	3	69.7%	0.72%
Abu-Ghanem et al., 2016 [31]	Hebrew	3	92.3%	97.3%
Möller et al., 2016 [19]	Swedish	3	98.5%	94.1%
Giraldo-Cadavid et al., 2016 [34]	Spanish (Colombia)	2	93.6%	36.4%
Printza et al., 2018 [22]	Greek	3	−	−
Järvenpää et al., 2021 [29]	Finnish	3	0.94%	0.96%

## Data Availability

All data generated or analyzed during this study are stored and pseudonymized by the corresponding author. Other questions can be directed to her.
